# Millet Disease

**Published:** 1893-12

**Authors:** T. D. Hinebauch


### MILLET DISEASE.

By T. D. Hinebauch, M.S., V.S.

HORSE HARRY.—(Concluded from page 322.)

| Date. | Nitrogen. | Total Solids. | Ash. |
|---|---|---|---|
| September 13...... | 1.55 | 9.50 | 2.05 |
| " 17...... | 1.58 | 11.65 | 2.90 |
| " 21...... | 1.16 | 10.08 | 1.88 |
| " 25...... | .96 | 5.58 | 1.90 |
| October 4........ | 1.35 | 7.48 | |

Carrying out the percentages, basing the calculations on the amount of urine collected, we find the following:

| Date. | Nitrogen. | Total Solids. | Ash. | Amt. of Urine. |
|---|---|---|---|---|
| | adv. | | | |
| September 13................ | 4.4 oz. | 1 lb. 11 oz. | 6 oz. | 17 lb. 14 oz. |
| " 17................ | 3.3 oz. | 1 lb. 9.1 oz. | 5.8 oz. | 13 lb. 8 oz. |
| " 21................ | 3.9 oz. | 2 lb. 1.8 oz. | 6 3 oz. | 21 lb. 0 oz. |
| " 25................ | 4.1 oz. | 1 lb. 7.7 oz. | 8.1 oz. | 26 lb. 11 oz. |
| October 4.................. | 3 8 oz. | 1 lb. 5.4 oz. | | 17 lb. 14 oz. |

We thus see that the amount of the different ingredients vary from day to day, and are not as might be supposed nearly constant. In other words, if the amount of urine increases the total increase is not alone of its water, but also of the other constituents as well.

If we take September 13th and October 4th, when the amount of urine was the same, and compare the total amount of nitrogen, we get a difference of .6 oz.

Yet we find that the proportions of nitrogen to total solids are the same, thus: 4.4 oz : 27 oz.=3.8 oz : 21¼ oz.

However, it is not to be relied upon, as the data are insufficient on which to base a calculation with any degree of accuracy.

